# The activity of the EMT suppressor GRHL2 is regulated by SUMOylation

**DOI:** 10.1016/j.jbc.2025.110662

**Published:** 2025-08-30

**Authors:** Sonja Santjer, Yang Xu, Sabine Riethdorf, Gerhard Schön, Johanna Neu, Klaus Pantel, Volker Assmann

**Affiliations:** 1 Institute of Tumor Biology, University Medical Center Hamburg-Eppendorf, Hamburg, Germany; 2 Institute for Medical Biometry and Epidemiology, University Medical Center Hamburg-Eppendorf, Hamburg, Germany

**Keywords:** Epithelial-mesenchymal transition (EMT), SUMOylation, posttranslational modification (PTM), transcription factor, oncogene, breast cancer, invasion, metastasis

## Abstract

The developmental transcription factor grainyhead-like 2 (GRHL2) has been attributed both tumor-suppressive and protumorigenic functions in a large variety of human cancers. Despite its fundamental role in cancer development and progression, mechanisms modulating expression or activity of GRHL2 in cancer cells still remain elusive. We identified several components of the SUMOylation machinery as candidate GRHL2 interactors using a yeast two-hybrid screening approach and a single major GRHL2 SUMOylation site at lysine residue 159. SUMOylation of GRHL2 at lysine 159 enhances its transcriptional activity and was found to be stimulated by phosphorylation of GRHL2 at threonine 164 by p38α/β MAPKs or by interaction with members of the PIAS family of SUMO E3 ligases. Additionally, structural analysis identified GRHL2 as an intrinsically disordered protein with a high propensity to misfold and to form aggresome-like structures in the nucleus, resulting in repression of GRHL2 transcriptional activity. Results obtained by immunohistochemical analysis of GRHL2 expression in primary breast cancers support an important role of GRHL2 subnuclear compartmentalization in breast carcinogenesis. Taken together, our results provide new insights into complex regulatory mechanisms governing GRHL2 activity in cancer cells.

The transcription factor grainyhead-like 2 (GRHL2) plays a crucial role in a variety of human cancers (1). GRHL2 is preferentially but not exclusively expressed in epithelial cells (2) and has emerged as a key regulator of epithelial cell morphogenesis and differentiation. It has long been recognized as a suppressor of the epithelial-to-mesenchymal transition (EMT), a morphogenetic program driving migration, invasion, and metastatic spread of tumors (3), through upregulation of epithelial junctional complex proteins, including E-cadherin and claudins 3 and 4, as well as by suppression of the EMT transcription factor zinc finger enhancer-binding protein 1 (ZEB1) (4, 5, 6, 7). Mechanistically, GRHL2 has been demonstrated to contain a small 13-amino motif capable of binding and inhibiting the histone acetyltransferase coactivator p300, thus preventing the transcriptional activation of p300-dependent target genes (*e.g.*, matrix metalloproteases) and EMT (8). Moreover, the interaction of GRHL2 with the histone methyltransferases KMT2C and KMT2D has been reported to promote mesenchymal-epithelial transition, ICAM-1 expression, and susceptibility of target cells to NK killing, suggesting that the functional interaction of GRHL2 with specific epigenetic modifiers is crucial for maintaining the epithelial phenotype and sensitivity to anoikis (8, 9). The role of a master regulator of EMT most convincingly has been demonstrated by the ability of GRHL2 to significantly impede transforming growth factor β- or Twist-induced and spontaneous EMT in breast cancer cells (4, 6).

Despite clear evidence of the tumor-suppressive role of GRHL2, it also has been reported to exhibit protumorigenic functions. For example, GRHL2 is known to epigenetically regulate human telomerase reverse transcriptase gene expression in oral squamous cell carcinoma cells, suggesting an important role in cellular immortalization (10, 11). It also suppresses death receptor (FAS and DR5) expression and evasion of cell death (12). Most importantly, however, GRHL2 has emerged as a regulator of cancer cell proliferation, and an oncogenic potential has been attributed to GRHL2 when overexpressed in NIH3T3 cells (6). In line with this, GRHL2 knockdown in colorectal cancer cells inhibited their proliferation and tumorigenesis in a mouse xenograft model (13). In luminal breast cancer, GRHL2 has been demonstrated to directly or indirectly regulate sets of genes involved in regulation of cell proliferation, thus substantiating a fundamental role of GRHL2 in regulation of tumor growth (14). Several recent studies further revealed an involvement of GRHL2 in hormone-dependent cancers. Here, GRHL2 has been shown to cooperate with androgen receptor in prostate cancer and with estrogen receptor α in breast cancer by acting as a pioneer factor facilitating chromatin accessibility. Therefore, GRHL2 represents a novel oncogenic cofactor stimulating transcriptional activity and signaling output of steroid hormone receptors (1, 15, 16, 17, 18, 19, 20, 21).

Numerous studies on GRHL2 mRNA or protein expression showed upregulation or downregulation of GRHL2 in a large variety of human cancers, possibly reflecting both tumor-suppressive and oncogenic functions of the GRHL2 transcription factor (1). It is generally assumed that GRHL2 activity may be dependent on the tumor type and/or stage of disease progression by regulating distinct target genes in diverse cancer types (1). In this respect, GRHL2 may resemble TGF-β/TGF-βR signaling which also is known to be largely context-dependent (22). Although the combined activation of transforming growth factor β/WNT signaling pathways has been demonstrated to induce ZEB1-dependent repression of GRHL2 expression and EMT (23), surprisingly little is known about mechanisms and signaling pathways regulating GRHL2 activity in cancer cells.

In this study, we identified several GRHL2-interacting proteins belonging to the SUMOylation pathway and identified SUMOylation as a novel GRHL2 regulatory mechanism. GRHL2 SUMOylation was found to be enhanced by phosphorylation and through interaction with PIAS proteins. Moreover, structural analysis identified GRHL2 as an intrinsically disordered protein (IDP) with a high propensity to misfold and to form aggresome-like structures, thus repressing GRHL2 transcriptional activity. Our findings provide new insights into complex regulatory networks controlling GRHL2 activity in cancer cells.

## Results

### Identification of members of the SUMOylation machinery as interactors of GRHL2

To uncover novel mechanisms regulating GRHL2 activity in cancer cells, a yeast two-hybrid screen of a human mammary gland adenocarcinoma MCF-7S1 complementary DNA (cDNA) library with the full-length human GRHL2 protein as bait was conducted. A total of 24 candidate interaction partners of GRHL2 could be identified using high-stringency quadruple dropout minimal medium (QDO)/X/A selective media. DNA sequencing and NCBI Blast search revealed that all isolated putative GRHL2-interacting proteins are members of the SUMOylation machinery and include SUMO-1 (n = 1), Ubc9 (n = 19), and truncated forms of PIAS2 (n = 1) and PIAS3 (n = 2) (Table 1). SUMOylation refers to the covalent attachment of small ubiquitin-like modifier (SUMO) to target proteins and involves a cascade of three enzymes, a SUMO E1-activating enzyme, the SUMO E2-conjugating enzyme Ubc9, and a SUMO E3 ligase. SUMOylation of proteins can drastically affect the subcellular localization, stability, ability to interact with other proteins, and activity of target proteins (24). The identification of several components of the SUMOylation cascade as putative interacting proteins strongly suggests a possible modification and regulation of GRHL2 by SUMOylation.

**Table 1:** GRHL2-interacting proteins identified using yeast two-hybrid screening

Gene symbol	Protein description	GeneBank accession number	Coded protein residues (complete)	Coded protein residues^a^ (retrieved)	No. of Occurences^b^
UBE2I	SUMO-conjugating enzyme UBC9 (variant 1)	NP_003336	158 aa	full-length	17
	SUMO-conjugating enzyme UBC9 (variant 4)	NP_919237	158 aa	full-length	2
SUMO1	Small ubiquitin-related modifier 1	NP_003343	101 aa	full-length	1
PIAS2	E3 SUMO-protein ligase PIAS 2 (PIAS xα)	NP_775298	572 aa	364-Stop	1
PIAS3	E3 SUMO-protein ligase PIAS 3	NP_006090	628 aa	382–540^c^	1
				435–540^c^	1

GRHL2, grainyhead-like 2; SUMO, small ubiquitin-like modifier.

a Residues indicate the amino acid residues of the protein fragments recovered in the yeast two-hybrid screen.

b Number of independent clones identified in the screen that encoded the same protein.

c Both PIAS 3 prey constructs contained sequences derived from exon 13, but lacked sequences encoded by the terminal exon 14. Instead, distinct sequences downstream of the terminal exon 14 were included in PIAS3 prey constructs, indicating that both PIAS3 prey proteins represent two distinct and novel PIAS3 isoforms.

### Lysine 159 is the major SUMOylation site in GRHL2 *in vivo*

**Figure 1:**
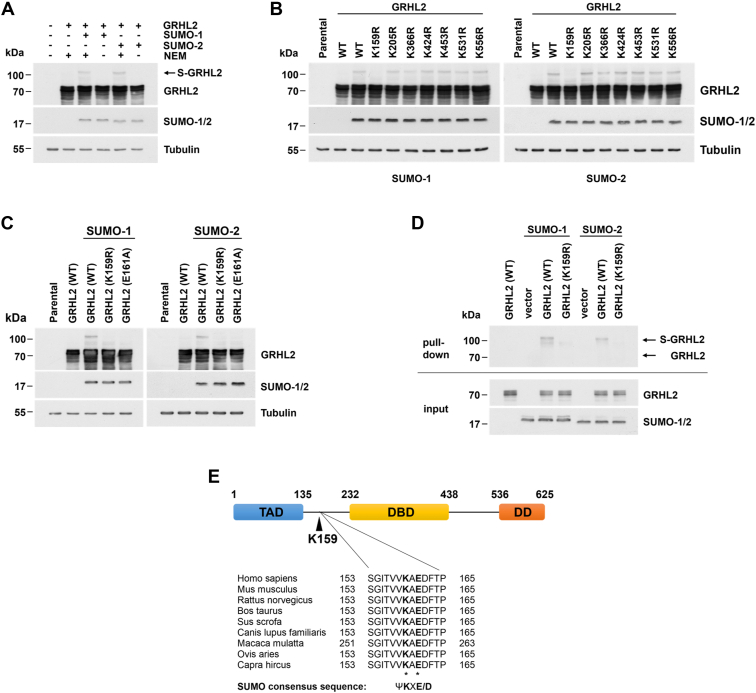
**Lysine 159 is the major SUMOylation site in GRHL2 *in vivo*.***A*, detection of SUMOylation of GRHL2 in transiently transfected COS-7 cells in the presence or absence of NEM and SUMO-1 and SUMO-2 by gel shift assay. *B*, identification of lysine 159 as the major SUMOylation site in GRHL2 as determined by gel shift analysis of GRHL2 (WT) and indicated GRHL2 mutant proteins in the presence or absence of SUMO-1 (*left*) or SUMO-2 (*right*). *C*, SUMOylation of GRHL2 occurs at a ΨKXD/E consensus motif for SUMOylation as demonstrated using GRHL2 (WT) and indicated GRHL2 mutant proteins in the presence or absence of SUMO-1 or SUMO-2 by gel shift analysis. *D*, SUMOylation of GRHL2 at lysine 159 was demonstrated by pull-down assay under denaturing conditions using His_6_-tagged SUMO-1 or SUMO-2 and nickel-affinity chromatography. Positions of non-SUMOylated (GRHL2) and SUMOylated GRHL2 (S-GRHL2) are marked with *arrows*. *E*, location of the major SUMOylation-site lysine 159 in the GRHL2 protein composed of an N-terminal transactivation domain (TAD), a central DNA-binding domain (DBD), and a C-terminal dimerization domain (DD) and sequence alignment of the region surrounding the SUMOylation site in GRHL2 orthologs. GRHL2, grainyhead-like 2; NEM, N-ethylmaleimide; SUMO, small ubiquitin-like modifier.

Covalent conjugation of SUMO proteins to substrates can cause shifts in the molecular weight by 10 to 40 kDa (24), thus enabling the detection of SUMOylated forms of a protein by gel shift assay. Using this approach, we identified in addition to the nonmodified GRHL2 protein with an apparent molecular weight of about 72 kDa, a GRHL2-specific band migrating at about 110 kDa in cell lysates of COS-7 cells transiently cotransfected with expression plasmids encoding GRHL2 and SUMO-1 or SUMO-2 (Fig. 1*A*). Detection of this slower migrating GRHL2-specific band was strictly dependent on coexpression of SUMO-1 or SUMO-2 and the presence of N-ethylmaleimide (NEM), a potent inhibitor of cysteine (thiol) proteases required for the preservation of the SUMOylated state of substrate proteins, in the lysis buffer. These results indicate that GRHL2 is modified by SUMOylation predominantly at a single lysine residue *in vivo*.

To identify high-probability SUMOylation sites in GRHL2, we performed a computational analysis of the GRHL2 protein sequence containing 53 potential acceptor sites for SUMOylation using ten different web-based SUMOylation prediction tools. Although a total of nineteen randomly distributed lysine residues were recognized as possible modification sites, only seven of these were predicted to be SUMOylation sites by at least two distinct computational approaches. To pinpoint the major SUMOylation site within GRHL2, candidate lysine residues (K159, K205, K366, K424, K453, K531, and K556) individually were replaced by arginine using site-directed mutagenesis, and WT and mutant GRHL2 proteins were then subjected to gel shift analysis. GRHL2 (WT) and all but the GRHL2 (K159R) mutant proteins were modified by SUMOylation, as indicated by the detection of the high-molecular weight GRHL2 proteins by Western blot analysis (Fig. 1*B*). This result was independent of whether SUMO-1 or SUMO-2 was coexpressed in COS-7 cells, thus substantiating a crucial role of residue K159 in the SUMOylation of the GRHL2 protein.

Lysine residues modified by SUMOylation are often embedded within a consensus motif ѰKxD/E, where Ѱ is a hydrophobic amino acid, K the SUMOylated lysine residue, x any amino acid, and D/E (aspartic/glutamic acid) (24). The major SUMOylation site in GRHL2 occurs in a sequence (VKAE^158-161^) which conforms to this consensus motif. To obtain additional evidence for an involvement of K159 in SUMOylation of GRHL2, the indispensable residue E161 within the putative GRHL2 consensus sequence was replaced by alanine, and SUMOylation of the GRHL2 (E161A) mutant was then analyzed by gel shift analysis (Fig. 1*C*). Disruption of the consensus sequence in GRHL2 at position E161 also completely abolished SUMOylation of GRHL2, thus confirming that high-molecular weight GRHL2 proteins represent SUMOylated forms of GRHL2 and are not caused by other posttranslational modifications requiring a lysine as an acceptor residue (*e.g.*, acetylation, ubiquitination).

To confirm our findings, pull-down experiments from COS-7 cells expressing GRHL2 (WT and mutant) proteins and His_6_-tagged SUMO-1/2 for the specific enrichment of SUMOylated proteins were performed. Cells were harvested using denaturing conditions, and SUMO-modified proteins were recovered by nickel-affinity chromatography. Using Western blot analysis, a selective enrichment of high-molecular weight GRHL2 adducts representing SUMOylated GRHL2 could be detected (Fig. 1*D*). The specificity of this approach was demonstrated by the absence of SUMOylated GRHL2 migrating at 110 kDa from cells expressing GRHL2 (K159R) proteins and of nonmodified GRHL2 migrating at about 72 kDa in all cell lysates.

These results strongly suggest that GRHL2 is modified by SUMOylation at residue K159 in the hinge region linking the N-terminal transactivation domain with the DNA-binding domain (DBD) of the GRHL2 transcription factor (Fig. 1*E*). Sequence alignment of the region surrounding the SUMOylation site in GRHL2 orthologs revealed a high degree of evolutionary conservation.

### SENP-1 and SENP-2 but not SENP-3, SENP-5, SENP-6, and SENP-7 deSUMOylated GRHL2

**Figure 2:**
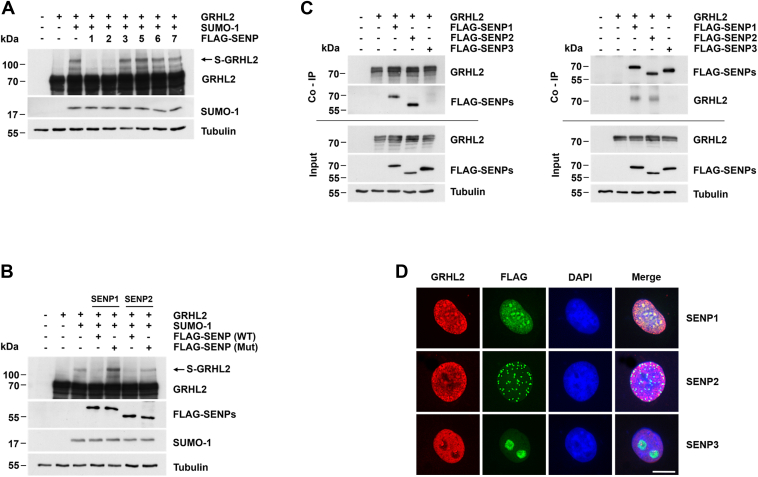
**DeSUMOylation of GRHL2 by SENP-1 and SENP-2.***A*, detection of deSUMOylation of GRHL2 by SENP-1 and SENP-2 but not SENP-3, SENP-5, SENP-6, or SENP-7 in transiently transfected COS-7 cells in the presence of SUMO-1 by gel shift assay. *B*, deSUMOylation of GRHL2 by SENP1 and SENP2 is dependent on their catalytic activity as demonstrated using WT or catalytically inactive FLAG-tagged SENP-1 and SENP-2 proteins (Mut) in gel shift assays. *C*, coimmunoprecipitation experiments using immobilized GRHL2 (*left*) or FLAG-SENPs (*right*) were employed to demonstrate a specific interaction of GRHL2 with SENP-1 and SENP-2, but not SENP-3. *D*, GRHL2 and SENP-1 and SENP-2 colocalize in the nucleoplasm of COS-7 cells as revealed by indirect double-immunofluorescence staining using anti-GRHL2 *(red*) and anti-FLAG M2 antibodies (*green*). Nuclei were visualized 4′,6-diamidino-2-phenylindole (*blue*). The scale bar represents 10 μm. GRHL2, grainyhead-like 2; SENP, sentrin/SUMO-specific protease; SUMO, small ubiquitin-like modifier.

SUMOylation is a highly dynamic process that can be reversed by sentrin/SUMO-specific proteases (SENPs) which in humans encompass six family members (SENP-1, SENP-2, SENP-3, SENP-5, SENP-6, and SENP-7) with distinct substrate specificities and subcellular localizations (25). To identify SENPs involved in GRHL2 deSUMOylation, GRHL2, SUMO-1, and FLAG-tagged SENPs were transiently expressed in COS-7 cells, and GRHL2 SUMOylation was assessed by gel shift assay. SENP-1 and SENP-2 significantly decreased SUMOylation of GRHL2, whereas other SENPs showed no effect (Fig. 2*A*). GRHL2 deSUMOylation was dependent on the enzymatic activity of SENP-1 and SENP-2, as catalytically inactive SENP-1 (C603S) and SENP-2 (C548S) failed to induce deSUMOylation of GRHL2 (Fig. 2*B*). GRHL2 also was found to specifically interact with SENP-1 and SENP-2 but not SENP-3 in coimmunoprecipitation assays using immobilized GRHL2 or in reciprocal experiments using an immobilized anti-FLAG antibody for enrichment of FLAG-tagged SENPs (Fig. 2*C*). Furthermore, immunofluorescence staining of COS-7 cells expressing GRHL2 and FLAG-tagged SENPs showed colocalization of GRHL2 with SENP-1 and SENP-2 in the nucleoplasm, whereas SENP-3 exclusively was localized to nucleoli of COS-7 cells (Fig. 2*D*). Taken together, these results established that GRHL2 is deSUMOylated primarily by SENP-1 and SENP-2.

### SUMOylation enhances transcriptional activity of GRHL2

**Figure 3:**
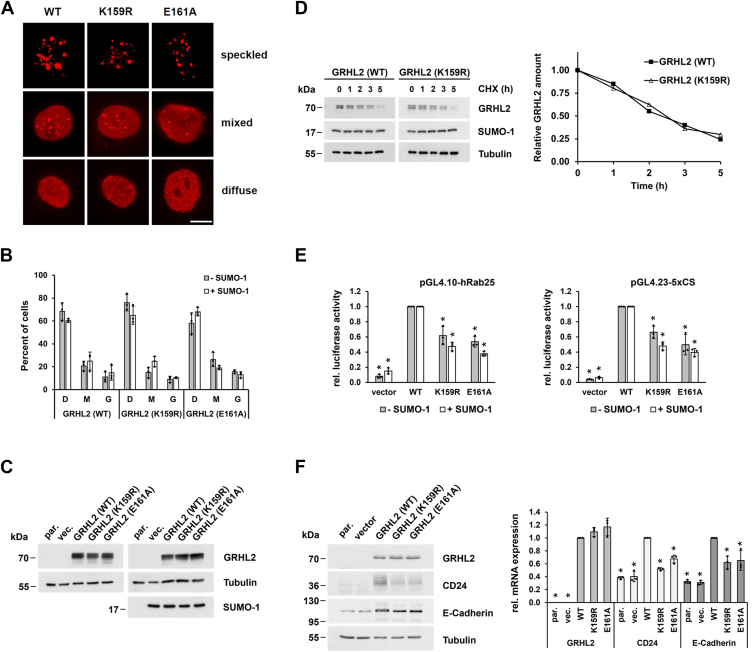
**SUMOylation affects activity but not stability or subnuclear localization of GRHL2.***A*, subcellular localization of WT or SUMOylation-deficient mutant GRHL2 proteins (K159R or E161A) in transiently transfected COS-7 cells was analyzed by indirect immunofluorescence analysis using anti-GRHL2 antibody (*red*). The scale bar represents 10 μm. *B*, quantification of diffuse (D), mixed (M), or granular (G) GRHL2 staining patterns in the absence (*filled bars*) or presence (*open bars*) of cotransfected SUMO-1. At least 100 cells were examined, and the mean ± SD values of three independent experiments are shown. *C*, expression levels of GRHL2 proteins in the absence (*left*) or presence (*right*) of cotransfected SUMO-1 in COS-7 cells determined by Western blot analysis. *D*, analysis of GRHL2 protein stability by CHX chase assay. Following treatment with CHX (50 μg/ml) for the indicated times, cell lysates were prepared and subjected to Western blot analysis. Results of one representative experiment (from three independent experiments) are shown *(left*). The graph illustrates the amount of GRHL2 proteins normalized by the amount of tubulin in the sample and plotted relative to the 0 h time point. The points in the graph represent the means of three independent experiments (*right*). *E*, transcriptional activity of GRHL2 proteins was determined using pGL4.10-hRab25 (*left*) or pGL4.23-5xCS (*right*) reporter plasmids in the absence (*filled bars*) or presence (*open bars*) of cotransfected SUMO-1. Luciferase activity from COS-7 cells transfected with GRHL2 (WT) was set arbitrarily at 1 for calculation of fold activation. Data represent the means ± SD values of three independent experiments performed in triplicate. *Asterisks* indicate statistically significant differences compared to GRHL2 (WT) using Student’s *t* tests (∗*p* < 0.05). *F*, expression of GRHL2 and its target genes CD24 and E-cadherin in MDA-MB-231 breast cancer cells expressing GRHL2 (WT) or mutant proteins was analyzed by Western blot (*left*) and qRT-PCR analysis (*right*). Expression of GRHL2, CD24, and E-cadherin mRNAs in GRHL2 (WT) expressing cells was set arbitrarily at 1, respectively. Data represent the means ± SD values of three independent experiments performed in triplicate. *Asterisks* indicate statistically significant differences compared to GRHL2 (WT) using Student’s *t* tests (∗*p* < 0.05). CHX, cycloheximide; GRHL2, grainyhead-like 2; qRT-PCR, quantitative real-time PCR; SUMO, small ubiquitin-like modifier.

Modification of proteins by SUMOylation can drastically alter functional properties of a target protein, such as its subcellular localization, stability, activation, and interactions with coregulatory molecules (24). We first analyzed the distribution of GRHL2 and its SUMOylation-deficient mutants in COS-7 cells by immunocytochemistry. Image analysis showed that all GRHL2 proteins shared an identical localization pattern and produced a speckled, mixed, or diffuse fluorescence staining of cells (Fig. 3*A*). Irrespective of whether SUMO-1 was coexpressed, quantification of staining results did not yield significant differences in the nuclear distribution, indicating that SUMOylation does not influence GRHL2 intranuclear compartmentalization (Fig. 3*B*).

Expression levels of GRHL2 (WT) and SUMOylation-deficient GRHL2 proteins in the presence or absence of cotransfected SUMO-1 and their half-life, as determined by cycloheximide (CHX) chase assay, appeared not to be affected by SUMOylation (Fig. 3, *C* and *D*), suggesting that SUMOylation does not play a major role in regulating GRHL2 stability.

We next assessed the potential effects of SUMOylation on the function of GRHL2 as a transcriptional regulator and developed two distinct luciferase-based reporter assays. For Rab25 promoter assay (pGL4.10-hRab25), the promoter of the human Rab25 gene, a known direct GRHL2 target gene (9, 26), was used, whereas the other reporter system (pGL4.23-5xCS) utilizes a synthetic GRHL2-sensitive enhancer containing five replicates of a GRHL2 consensus binding motif (AACCGGTT) upstream of a minimal promoter and the firefly luciferase gene (*luc2*). In GRHL2 (WT) transfected cells, the level of reporter activity was approximately 12-fold (pGL4.10-hRab25) and 25-fold (pGL4.23-5xCS) higher than in vector-transfected cells, respectively (Fig. 3*E*). Compared to GRHL2 (WT), the level of reporter activity was significantly lower in COS-7 cells expressing SUMOylation-deficient GRHL2 variants both in pGL4.10-hRab25 (approx. 61% for GRHL2 (K159R) and 54% for GRHL2 (E161A)) and pGL4.23-5xCS (approximately 66% for GRHL2 (K159R) and 50% for GRHL2 (E161A)) luciferase reporter assays. As expected, coexpression of SUMO-1 resulted in more pronounced differences between GRHL2 (WT) and its mutant proteins (pGL4.10-hRab25: approximately 48% for GRHL2 (K159R) and 38% for GRHL2 (E161A) and pGL4.23-5xCS: approximately 48% for GRHL2 (K159R) and 40% for GRHL2 (E161A)) (Fig. 3*E*).

To further substantiate these findings, GRHL2 (WT) or SUMOylation-deficient GRHL2 mutants were stably expressed in GRHL2-negative MDA-MB-231 breast cancer cells by means of retroviral gene transfer, and expression of GRHL2 and selected GRHL2 target genes CD24 and E-cadherin (CDH1) then was analyzed by Western blot and quantitative real-time PCR (qRT-PCR) analysis. Although SUMOylation-deficient GRHL2 proteins were expressed at a slightly higher level than GRHL2 (WT) proteins (about 1.1- or 1.2-fold, respectively), induction of CD24 and E-cadherin/CDH1 mRNA and protein expression still was significantly reduced compared to GRHL2 (WT) expressing cells (Fig. 3*F*). These results together with those obtained by two distinct luciferase reporter assays therefore strongly suggest that SUMOylation enhances activity of the GRHL2 transcription factor.

### SUMOylation of GRHL2 at K159 is augmented by phosphorylation at T164

**Figure 4:**
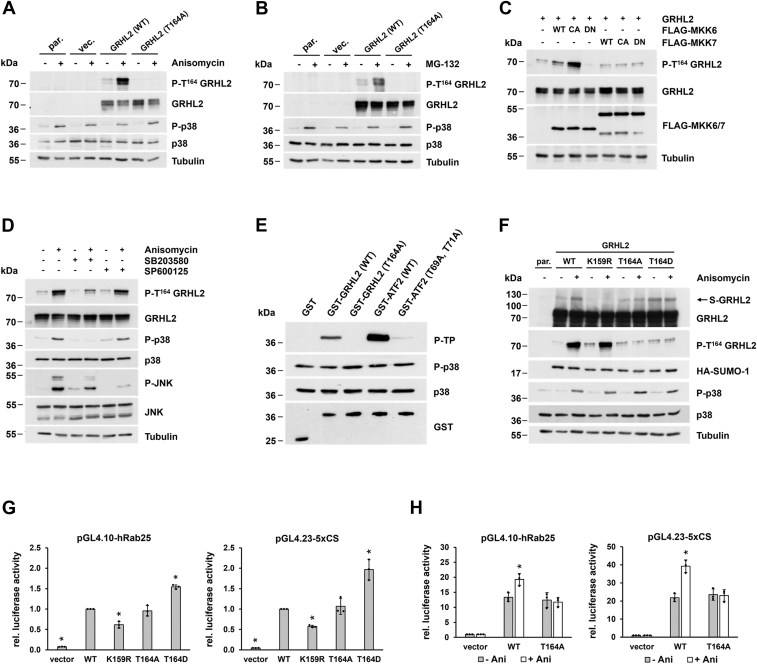
**Phosphorylation at T164 enhances SUMOylation at K159 and GRHL2 transcriptional activity.***A* and *B*, treatment of GRHL2-transfected COS-7 cells with anisomycin (25 μg/ml, 1.5 h) or with the proteasome inhibitor MG-132 (20 μM, 4 h) induced phosphorylation of GRHL2 at T164 as determined by Western blot analysis using a phospho-GRHL2 (T164) antibody. *C*, phosphorylation of GRHL2 at residue T164 is mediated by MKK6 but not MKK7, as demonstrated by transfection of WT, constitutively active (CA), or dominant-negative (DN) versions of FLAG-tagged MKK6 or MKK7 into GRHL2-expressing COS-7 cells. *D*, phosphorylation of GRHL2 at residue T164 specifically can be inhibited by pretreatment with p38 MAPK inhibitor SB203580 (10 μM, 1 h) but not SAPK/JNK inhibitor SP600125 (50 μM, 40 min) in anisomycin-stimulated (25 μg/ml for 1.5 h) GRHL2-expressing COS-7 cells. *E*, phosphorylation of GRHL2 at residue T164 was demonstrated by *in vitro* kinase assay using GST, GST-GRHL2 (WT and T164A mutant), and GST-ATF2 (WT and T69A, T71A double-mutant) fusion proteins and activated p38α MAPK, as visualized by Western blot analysis. *F*, SUMOylation of GRHL2 at residue K159 is stimulated by anisomycin-induced GRHL2 (T164) phosphorylation or by the introduction of a phosphomimetic aspartate residue at position 164 of the GRHL2 sequence as determined by gel shift analysis. *G*, transcriptional activity of GRHL2 is enhanced by introducing a phosphomimetic aspartate residue at position 164 of the GRHL2 sequence as determined using pGL4.10-hRab25 (*left*) or pGL4.23-5xCS (*right*) based luciferase reporter assays. Luciferase activity from COS-7 cells transfected with GRHL2 (WT) was set arbitrarily at 1 for calculation of fold activation. Data represent the means ± SD values of three independent experiments performed in triplicate. *Asterisks* indicate statistically significant differences compared to GRHL2 (WT) using Student’s *t* tests (∗*p* < 0.05). *H*, stimulation of cells with anisomycin (Ani) (25 μg/ml for 1.5 h) resulted in increased transcriptional activity of GRHL2 (WT) but not GRHL2 (T164A) proteins in luciferase reporter assays. Luciferase activity from COS-7 cells transfected with empty vector was set arbitrarily at 1 for calculation of fold activation. Data represent the means ± SD values of three independent experiments performed in triplicate. *Asterisks* indicate statistically significant differences compared to the corresponding unstimulated cells using Student’s *t* tests (∗*p* < 0.05). GRHL2, grainyhead-like 2; GST, glutathione-S transferase.

Residues flanking the SUMOylation consensus sequence, which represents the binding site for the only SUMO E2-conjugating enzyme Ubc9 (27), can significantly stimulate or repress target-specific SUMOylation (24), and extended variants of the consensus motif including the negatively charged amino acid–dependent SUMOylation motifs and the phosphorylation-dependent SUMOylation motifs were identified (28, 29). The SUMOylation site in GRHL2 conforms to the PSDM consensus motif (ѰKXEXXSP) and includes, in addition to a negatively charged residue (aspartate 162), a phosphorylatable threonine-proline motif downstream of the SUMOylation consensus sequence (^158^VKAEDFTP^165^). To investigate whether GRHL2 contains such a bipartite motif, polyclonal phospho-GRHL2 (T164) antibodies were generated which, however, showed only very weak reactivity in Western blot analysis. Since results obtained using various phosphorylation prediction servers hinted toward a possible phosphorylation of GRHL2 at position 164 by p38 MAPK and SAPK/JNK family members, we specifically induced activation of these kinases by treatment of cells with anisomycin and observed a striking increase in GRHL2 phosphorylation which was strictly dependent on the presence of phosphorylatable threonine 164 in the GRHL2 sequence (Fig. 4*A*). Other experimental conditions known to induce activation p38 MAPKs and SAPK/JNKs, such as application of proteotoxic stress by treatment of cells with the proteasome inhibitor MG-132, produced the same effect (Fig. 4*B*). To determine which MAPK family member preferentially modifies GRHL2 at threonine 164, WT, constitutively active (CA), or dominant-negative versions of FLAG-tagged MKK6 or MKK7 kinases, which specifically activate either p38 MAPKs (MKK6) or SAPK/JNKs (MKK7) (30), were introduced into GRHL2-expressing COS-7 cells. Western blot analyses showed that MKK6 (WT) and MKK6 (CA) but not the corresponding versions of MKK7 stimulated phosphorylation of GRHL2, suggesting an involvement of p38 MAPK isoforms in the posttranslational modification of GRHL2 at position T164 (Fig. 4*C*).

To identify p38 MAPK family members (α, β, γ, or δ) mediating GRHL2 (T164) phosphorylation, GRHL2-expressing COS-7 cells were treated with p38 MAPK or SAPK/JNK inhibitors followed by stimulation with anisomycin. Pretreatment with the p38 MAPK inhibitor SB203580 but not SAPK/JNK inhibitor SP600125 (negative control) significantly reduced phosphorylation of GRHL2 at position 164 (Fig. 4*D*). Owing to structural differences in the ATP binding pocket of p38 MAPKs, the SB203580 compound specifically blocks the activity of p38α/β MAPKs, but not γ/δ isoforms (31). To further verify an involvement of p38α/β MAPKs, *in vitro* kinase assays using purified glutathione-S transferase (GST) fusion proteins (GST, GST-GRHL2 (T118-R209), and GST-ATF2 (M19-L114) and mutant proteins thereof) were performed. We observed that the closely related p38α/ß MAPK isoforms phosphorylated GRHL2 at threonine 164 to a comparable extent. Representative results obtained for p38α MAPK are shown in Figure 4*E*.

Next, we investigated the potential effects of phosphorylation at threonine 164 on SUMOylation of GRHL2 and introduced two distinct single-site point mutations in GRHL2 by replacing the phosphorylatable residue T164 by alanine or a phosphomimetic aspartic residue, respectively. GRHL2 (WT and mutants) were then analyzed by gel shift assay following stimulation with anisomycin. Treatment of cells with anisomycin significantly increased SUMOylation of GRHL2, an effect which could not be seen using the SUMOylation-deficient GRHL2 (K159R) or the nonphosphorylatable GRHL2 (T164A) mutants (Fig. 4*F*). GRHL2 (T164D) proteins with a phosphomimetic aspartic acid at position 164 displayed increased SUMOylation in gel shift assays which could not be further augmented by anisomycin treatment. These results clearly indicate that phosphorylation of GRHL2 at threonine 164 promotes SUMOylation at position lysine 159.

To assess the potential effects of increased SUMOylation induced by phosphorylation of threonine 164 on GRHL2 transcriptional activity, we again employed luciferase-based reporter assays. In cells transfected with GRHL2 (T164D) cDNA harboring a phosphomimetic aspartic acid at position 164, the level of reporter activity was approximately 1.54-fold (pGL4.10-hRab25) and 1.97-fold (pGL4.23-5xCS) higher than in GRHL2 (WT)-transfected cells, respectively, whereas the level of reporter activity was largely unchanged in COS-7 cells expressing the nonphosphorylatable GRHL2 (T164A) mutant (Fig. 4*G*). Likewise, stimulation of cells with anisomycin resulted in increased GRHL2 transcriptional activity in both luciferase-based reporter assays which was dependent on the presence of the phosphorylatable residue threonine T164A (Fig. 4*H*). Collectively, these results strongly suggest that increased SUMOylation induced by phosphorylation of threonine 164 significantly enhances GRHL2 transcriptional activity.

### Stimulation of SUMOylation of GRHL2 by PIAS proteins

**Figure 5:**
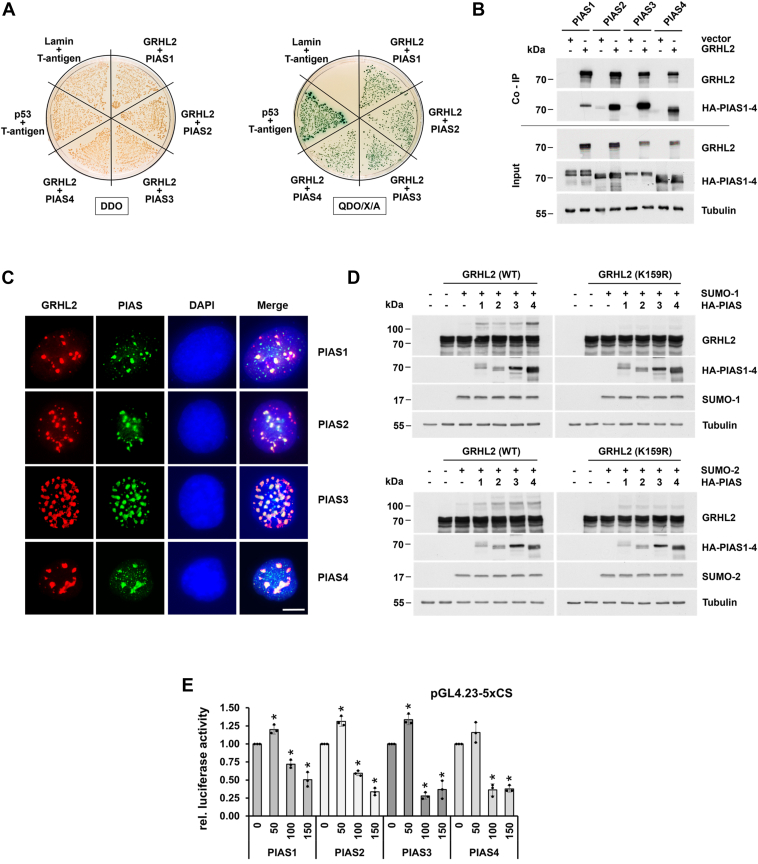
**SUMOylation of GRHL2 is augmented by PIAS proteins.***A*, interaction of GRHL2 with PIAS1-4 proteins was confirmed by cotransformation of yeast with GRHL2 bait and PIAS1-4 prey plasmids and plating on double dropout minimal medium *(left*) or high stringency quadruple dropout minimal medium/X/A selective media (*right*). Cotransformation of p53 or lamin bait plasmids with large T-antigen prey plasmids served as positive and negative controls in these experiments, respectively. *B*, pull-down experiments using Twin-Strep-Tag-GRHL2 and HA-tagged PIAS proteins and MagStrep “type 3” XT magnetic beads revealed a specific interaction of GRHL2 with all four PIAS proteins in GRHL2-transfected but not empty vector transfected cells. *C*, GRHL2 and PIAS proteins colocalize in granular structures in the nucleoplasm of COS-7 cells as determined by indirect immunofluorescence staining using anti-GRHL2 *(red*) and anti-HA antibodies (*green*). Nuclei were visualized with 4′,6-diamidino-2-phenylindole (*blue*). The scale bar represents 10 μm. *D*, PIAS1-4 proteins augment SUMOylation of GRHL2 (WT) but not GRHL2 (K159R) mutant proteins in the presence of cotransfected SUMO-1 (*top*) or SUMO-2 (*bottom*) as demonstrated by gel shift assay. *E*, repression of GRHL2 transcriptional activity by PIAS1-4 proteins was demonstrated using the pGL4.23-5xCS based luciferase reporter assay without coexpressed SUMO proteins. Luciferase activity from COS-7 cells lacking PIAS1-4 proteins (0 ng) was set arbitrarily at 1 for calculation of fold activation each. Data represent the means ± SD values of three independent experiments performed in triplicate. *Asterisks* indicate statistically significant differences compared to luciferase activity measured in COS-7 cells lacking PIAS1-4 using Student´s *t* tests (∗*p* < 0.05). GRHL2, grainyhead-like 2; SUMO, small ubiquitin-like modifier.

Human PIAS proteins are encoded by four distinct genes, namely PIAS1, PIAS2 (PIASx), PIAS3, and PIAS4 (PIASy) (32). Results obtained by employing an unbiased yeast two-hybrid approach suggest a possible interaction of GRHL2 with at least two members of the PIAS family of SUMO E3 ligases (PIAS2 and PIAS3) (see Table 1). Using one-to-one yeast transformation experiments, GRHL2 was found to interact with all four members of the PIAS family of SUMO E3 ligases in a yeast two-hybrid assay using high stringency QDO/X/A selective medium. These interactions were comparable in strength but were slightly weaker than the interaction between p53 and large T-antigen (positive control) (Fig. 5*A*). These results were confirmed by pull-down experiments using immobilized GRHL2 (Fig. 5*B*). Colocalization analyses by double indirect immunofluorescence staining showed that all PIAS proteins unequivocally and almost completely induced a change in GRHL2 subnuclear localization, resulting in colocalization of GRHL2 and PIAS proteins in granular structures (Fig. 5*C*). The PIAS-induced aggregation of GRHL2 appears to be largely independent of SUMOylation as cotransfection of SUMOylation-deficient GRHL2 (K159R) proteins with PIAS proteins or GRHL2 (WT) with catalytically inactive PIAS proteins also was found to induce GRHL2 aggresome formation (data not shown).

To further substantiate a functional interaction between GRHL2 and PIAS proteins, cell lysates from COS-7 cells coexpressing GRHL2 (WT or K159R mutant), individual PIAS proteins, and SUMO-1 or SUMO-2 were subjected to gel shift assay. All four PIAS proteins were found to significantly increase SUMOylation of GRHL2 (Fig. 5*D*). This effect was dependent on the presence of the acceptor lysine for SUMOylation of GRHL2 (K159) and could be detected with SUMO-1 and SUMO-2. These findings indicate that PIAS proteins display SUMO E3 ligase activity on GRHL2 and therefore represent novel coregulators of GRHL2 by promoting SUMOylation at lysine 159.

To define the potential effects of increased SUMOylation induced by members of the PIAS family of SUMO E3 ligases, COS-7 cells cotransfected with expression plasmids encoding GRHL2 (WT) (100 ng) and increasing amounts of PIAS1-4 proteins (0, 50, 100, and 150 ng) were subjected to luciferase reporter assays using the pGL4.23-5xCS reporter plasmid. All PIAS proteins at relatively low concentrations (50 ng) were found to slightly stimulate GRHL2 transcriptional activity, whereas at higher concentrations (100 and 150 ng) a repressive effect compared to luciferase activity measured in COS-7 cells lacking PIAS1-4 (0 ng) was detected each (Fig. 5*E*).

### GRHL2 is an IDP with a high propensity for aggregation

**Figure 6:**
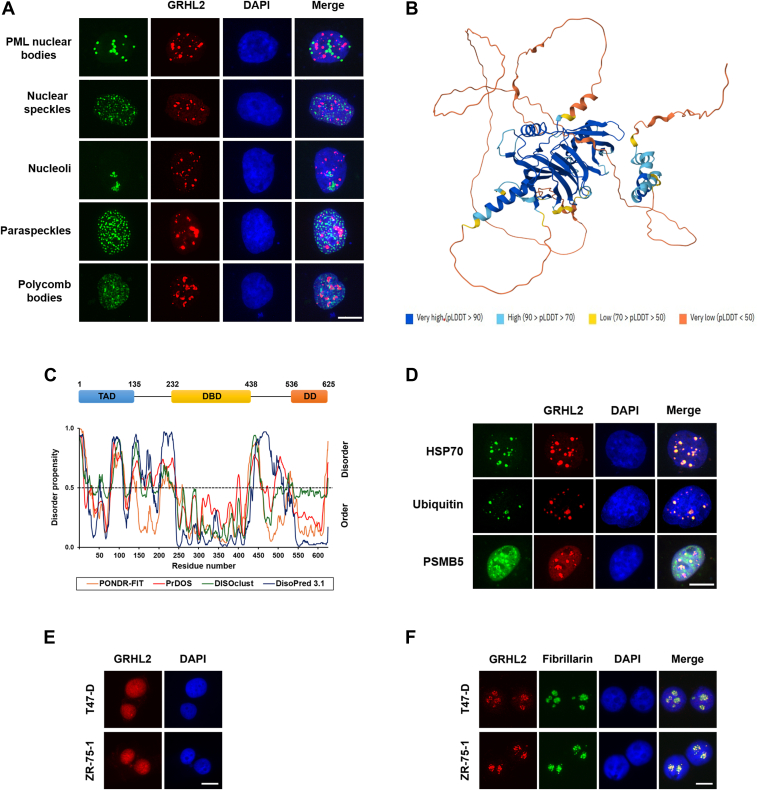
**GRHL2 is an intrinsically disordered protein with a high propensity for aggregation.***A*, GRHL2 did not colocalize with various nuclear sub-bodies as revealed by indirect immunofluorescence staining using antibodies recognizing GRHL2 (*red*) and antibodies recognizing defining components of the indicated nuclear substructures (promyelocytic leukemia, SC-35, fibrillarin, PSPC1/PSP-1, and EZH2) (*green*). *B*, AlphaFold2 algorithm was used to predict the overall structure of *Homo sapiens* GRHL2 (UniProt: Q6ISB3). Each residue of the protein is colored corresponding to a per-residue model confidence score (predicted local-distance difference test) between 0 and 100, with higher scores reflecting better confidence. Regions with a predicted local-distance difference test <50 have a ribbon-like appearance and may represent unstructured sequences. *C*, prediction of intrinsically disordered regions in GRHL2 using PrDOS (*red*), PONDR-FIT (*orange*), DISOclust (*green*), and DisoPred 3.1 (*blue*) servers. A probability value higher than 0.5 indicates a tendency to be unfolded (*black dashed line*). The domain structure of GRHL2 and the indicated location of domains are schematically depicted on *top*. *D*, GRHL2 granular structures are enriched with aggresomal marker proteins as revealed by indirect immunofluorescence staining of COS-7 cells using the standard immunofluorescence staining protocol with an anti-GRHL2 antibody (*red*) and antibodies specific for HSP70, ubiquitin, and PSMB5, respectively (*green*). *E*, the distribution of GRHL2 in breast cancer cell lines was determined by indirect immunofluorescence staining using the standard immunofluorescence staining protocol with a GRHL2-specific antibody (*red*). *F*, GRHL2 aggresome-like structures and colocalization with nucleoli were visualized in breast cancer cells using a detergent pre-extraction double-immunofluorescence protocol and antibodies recognizing GRHL2 (*red*) or fibrillarin (*green*). *A* and *D-F*, nuclei were counterstained with 4′,6-diamidino-2-phenylindole (*blue*). The scale bar represents 10 μm. GRHL2, grainyhead-like 2; SUMO, small ubiquitin-like modifier.

We hypothesized that the repressive effect of PIAS proteins on GRHL2 transcriptional activity could be related to the PIAS-induced accumulation of GRHL2 proteins in granule-like structures and investigated a possible translocation of GRHL2 into any of the many known nuclear bodies of the cell. However, indirect immunofluorescence staining with antibodies recognizing defining components of diverse nuclear substructures did not provide evidence for an accumulation of GRHL2 in known nuclear substructures (Fig. 6*A*).

We next considered the possibility that GRHL2 may belong to the family of IDPs which are known to have a high propensity to form aggresome-like structures (33). Analysis of the overall structure of GRHL2 using AlphaFold2 revealed the presence of extended unstructured regions in the GRHL2 protein sequence depicted as ribbon-like structures with a very low predicted local-distance difference test (Fig. 6*B*). Results obtained using four distinct web tools for disorder prediction confirmed that, except for the highly structured DNA binding (DBD) and dimerization domains, the GRHL2 sequence mostly is predicted to be disordered (disorder prediction probability value > 0.5) with a disorder content ranging from 24.96% (PONDR-FIT) to 39.04% (PrDOS) (mean 33.06%) (Fig. 6*C*). Since aggresome-like structures are known to concentrate, apart from the misfolded substrate proteins, Hsp70 and other chaperones, and a degradation machinery comprising ubiquitin and the 20S proteasome (33), indirect immunofluorescence staining experiments were performed. The results shown in Figure 6*D* demonstrate enrichment of GRHL2 granule-like structures with aggresomal marker proteins.

We next analyzed the distribution of GRHL2 in breast cancer cell lines by indirect immunofluorescence staining and found that GRHL2 mostly was dispersed in the nucleoplasm of cells (Fig. 6*E*). We noticed, however, that the distribution of GRHL2 was not homogenous, raising the possibility that GRHL2 aggresome-like structures might be masked by the presence of soluble GRHL2 proteins in the nucleoplasm. Therefore, we performed additional immunocytochemical analyses using an alternative protocol which involved a detergent pre-extraction of soluble proteins prior to fixation of cells. Using this approach, we readily could detect GRHL2 granular structures in the nucleus of breast cancer cells (Fig. 6*F*). In contrast to COS-7 cells, GRHL2 granules almost completely occurred in nucleoli, as revealed by double-immunofluorescence staining of cells using antibodies recognizing GRHL2 and fibrillarin, a marker for nucleoli (and Cajal bodies). Overall, our findings led us to conclude that aggregation is physiologically relevant and leads to functional inactivation of the IDP GRHL2 by preventing the GRHL2 transcription factor from effectively binding to target gene promoters.

### Associations of GRHL2 subnuclear distribution with histopathological variables

**Figure 7:**
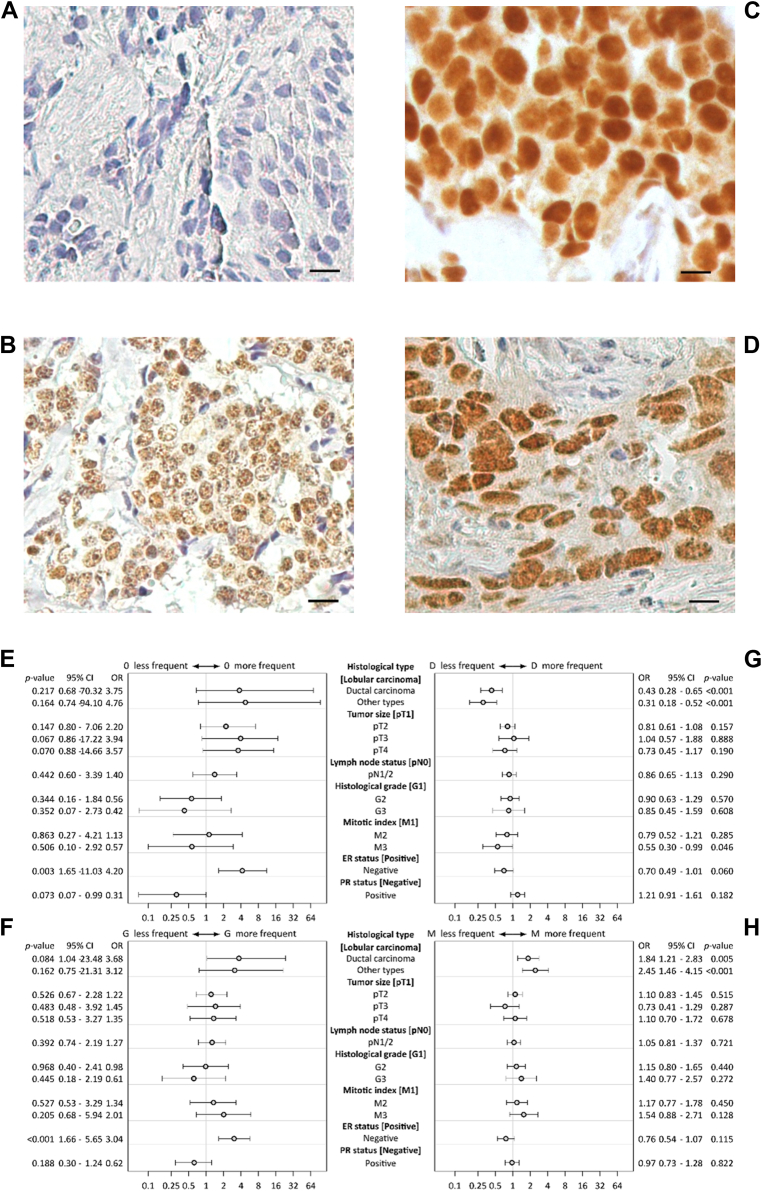
**Subnuclear distribution of GRHL2 in breast cancer.***A–D*, GRHL2 staining patterns representing negative, granular, diffuse, and mixed subnuclear distribution, respectively. Original magnification, x400; the scale bar represents 10 μm. *E–H*, forest plots showing associations of distinct subnuclear staining patterns (negative: 0, granular: G, diffuse: D, and mixed: M) with histopathological parameters, respectively. CI, confidence interval; ER, estrogen receptor; GRHL2, grainyhead-like 2; OR, odds ratio; PR, progesterone receptor; SUMO, small ubiquitin-like modifier.

The subnuclear distribution of GRHL2 in primary breast cancers was analyzed using a tissue microarray with more than 2000 specimens and a GRHL2-specific antibody. Staining results showed that 2.9% of tumors were GRHL2 negative, 41.6% presented with predominantly diffuse, 7.1% with predominantly granular, and 48.4% with mixed (diffuse and granular) staining. Representative staining results are shown in Figure 7, *A*–*D*. Using logistic regression models, the association between distinct GRHL2 subnuclear patterns and histopathological parameters was examined for tissue samples with all data available (n = 1075) (Table S1), and estimated results are displayed by forest plots (Fig. 7, *E*–*H*). Significant correlations between GRHL2 negativity (*p = 0.003,* Fig. 7*E*) or a granular distribution (*p < 0.001,* Fig. 7*F*) with negative estrogen receptor status were found. A diffuse GRHL2 distribution was significantly less frequent in ductal carcinomas (*p < 0.001*) and other types (*p < 0.001*) than in lobular carcinomas (Fig. 7*G*). The number of cases with diffuse GRHL2 distribution was lowest in tumors with the highest mitotic index (*p < 0.046*). Compared to lobular, ductal carcinomas (*p = 0.005*) and other types (*p* < 0.001) more frequently displayed a mixed GRHL2 immunostaining (Fig. 7*H*). GRHL2 subnuclear distribution did not correlate with disease-specific (*p* = 0.232, Fig. S1*A*) but with overall survival (*p* < 0,001, Fig. S1*B*). Multivariate analysis, however, did not suggest that GRHL2 subnuclear distribution is an independent prognostic factor for overall survival (Fig. S1*C*).

## Discussion

Despite overwhelming evidence for both tumor-suppressive and protumorigenic roles of the GRHL2 transcription factor in a large variety of human tumors, mechanisms modulating expression or activity of GRHL2 in cancer cells still are largely unknown. We identified several components of the SUMOylation cascade as candidate GRHL2 interactors using a yeast two-hybrid screening approach and a single major GRHL2 SUMOylation site at lysine residue 159. Mechanistic studies revealed that SUMOylation predominantly enhances transcriptional activity of GRHL2. Although SUMOylation of transcription factors appears to have a general tendency to repress transcriptional activity, a growing number of transcriptional regulators were reported to be activated by SUMOylation (33). Since the effect of SUMO modification on transcriptional activity can depend on the target gene promoter and/or cellular context, we employed two distinct luciferase-based reporter assays and MDA-MB-231 breast cancer cells as a model system to demonstrate that SUMO modification enhances GRHL2 transcriptional activity, possibly through induction of structural changes, enhancement or inhibition of protein–protein interactions, or by influencing GRHL2 protein solubility. In this study, we show that SUMO conjugation or deconjugation specifically mediated by SENP-1 and SENP-2 proteases represents a novel regulatory mechanism of GRHL2-dependent transcriptional activity in cancer cells.

We further characterized two distinct mechanisms controlling SUMOylation-dependent GRHL2 transcriptional activity, including phosphorylation of GRHL2 at threonine 164 by p38α/β MAPKs. This finding is in line with the notion that posttranslational modifications (phosphorylation, ubiquitinylation, acetylation, and methylation) proximal to canonical SUMOylation consensus motifs can stimulate or repress SUMOylation. In the case of phosphorylation, it is thought that the negative charge conferred by phosphorylation and/or acidic residues facilitates SUMO conjugation of the target lysine by enhancing recruitment of the SUMO E2 ligase Ubc9 (28, 34). The major SUMOylation site in GRHL2 fully conforms to the phosphorylation-dependent SUMOylation motif (29) and includes a phosphorylatable threonine-proline motif downstream of the SUMOylation consensus sequence. Phosphorylation of GRHL2 at residue T164 significantly enhanced SUMOylation and GRHL2 transcriptional activity. Co-modification of target molecules with SUMO and proline-directed phosphorylation frequently occurs in the SUMOylone and therefore is recognized as a major cellular signaling mechanism (35).

SUMO modification of GRHL2 also is controlled by the physical and functional interaction with members of the PIAS family of SUMO E3 ligases, pleiotropic factors which interact and regulate a large variety of distinct proteins, especially transcriptional regulators (32). Like several other transcription factors, multiple PIAS proteins interact with GRHL2 and promote its SUMOylation *in vivo*. Contrary to expectations, however, an increased SUMOylation of GRHL2 by PIAS proteins caused decreased rather than enhanced GRHL2 transcriptional activity. Taken that PIAS proteins have a reputation of exerting unexpected effects on target molecules, this finding is not surprising. Well-known examples include various members of the steroid hormone receptor family where PIAS proteins positively or negatively modulate the ligand-induced transactivation potential depending on the receptor type, cell system, or promoter (32). As demonstrated for the ETS family member FLI-1 (36), the functional outcome of an interaction of a target molecule with PIAS proteins also can be severely influenced by a PIAS-dependent targeting to specific nuclear substructures, especially promyelocytic leukemia nuclear bodies. PIAS proteins uniformly dragged GRHL2 into granular structures which shared characteristics with aggresomes. In line with this, structural analysis revealed that GRHL2 belongs to a growing number of IDPs which are known to have a high propensity to misfold and aggregate in cells (37). Thus, PIAS proteins appear to elicit an unexpected misfolded protein response, resulting in the formation of nucleoplasmic aggresome-like structures and sequestration of GRHL2 away from its target gene promoters, thus neutralizing its transcriptional activity. Although it is unclear as to whether other coregulatory molecules elicit similar responses, our findings have broad implications for experimental design and the interpretation of results when studying effects of coregulatory molecules on the activity of GRHL2 and possibly other members of the grainyhead family of transcription factors.

The formation of GRHL2 aggresome-like structures occurred also in breast cancer cells and indicates participation of GRHL2 proteins in a quality control pathway described recently (38). Nuclear proteins upon cell stress immerse into the liquid-like granular component phase of the nucleolus to prevent their irreversible aggregation and to render them competent for refolding by Hsp70 and other molecular chaperones. Upon recovery from stress, mobility of misfolded proteins is restored, allowing their exit from the nucleolus and refolding or proteasomal degradation in the nucleoplasm. The capacity of the nucleolus to store misfolded proteins is limited, and prolonged stress causes formation of aggregates with amyloid-like properties of proteins in the nucleoplasm. It can be envisaged that GRHL2 overexpression exceeds the maximum capacity of the protein quality control system in the nucleus, thus resulting in accumulation of GRHL2 aggregates in the nucleoplasm. The formation of GRHL2 aggresome-like structures results in its functional inactivation, hence adding an additional layer of complexity to the regulation of the GRHL2 transcription factor in cancer cells.

Associations between GRHL2 expression and clinical outcome in different cancer types reported so far mostly, but not exclusively, are based on differences in GRHL2 expression determined using publicly available omics datasets and, not surprisingly, in many cases even yielded contradictory results (reviewed in (1)). This strongly suggests that different cancer cohorts, methodology (*e.g.*, GRHL2 mRNA *versus* protein analyses), or selection of distinct survival analysis endpoints severely affect the outcome of GRHL2 association analyses. Considering the many known limitations of gene expression analyses using omics-based datasets and the heterogeneous subnuclear distribution in breast cancer cells, more elaborated GRHL2 protein expression analyses are needed. Our findings take account of distinct GRHL2 expression patterns reflecting different GRHL2 activation states but clearly need to be confirmed using distinct breast and other cancer cohorts.

Oncogenic EMT plays a crucial role in cancer progression, allowing cancer cells to rapidly adapt to various intrinsic or microenvironmental changes by switching from epithelial (E) to mesenchymal phenotype (M) or by retaining a metastable, hybrid phenotype (hybrid E/M) (3). EMT/mesenchymal-epithelial transition is mediated by modulating regulatory networks of a set of transcription factors which either promote or suppress EMT. Mooney and co-workers showed that key regulators of EMT (*e.g.*, ZEB1, SNAI1, and OVOL1/2) represent IDPs and hypothesized that it is their conformational flexibility which enables the formation of highly dynamic protein interaction networks and phenotypic plasticity of cancer cells (39). Although the concept of linking structural flexibility of IDPs with phenotypic switching is very interesting, direct experimental evidence supporting this hypothesis is still limited. Our findings underscore the importance of conformational dynamics of IDPs in regulation of phenotypic plasticity in cancer invasion and metastasis.

## Experimental procedures

### Yeast two-hybrid screening

The yeast two-hybrid screen was performed using the MATCHMAKER Gold Yeast Two-Hybrid System (Clontech) according to the manufacturer’s instructions. The bait plasmid was generated by inserting a PCR-amplified fragment encoding full length human GRHL2 (UniProt accession Q6ISB3) into the pGBKT7 plasmid. The plasmid was transformed into the *Saccharomyces cerevisiae* strain Y2H Gold and was tested for autoactivation and toxicity. Subsequently, the mammary gland adenocarcinoma MCF-7S1 DUALhybrid cDNA library (Dualsystems) was transformed into yeast using the polyethylene glycol/lithium acetate method as described (40). For library screening, about 4 × 10^6^ yeast transformants were plated onto 150-mm plates containing SD/-Leu/-Trp/X-a-Gal/AbA (double dropout minimal medium/X/A) and incubated for 5 days at 30 °C, and then the surviving clones were further selected using more stringent SD/-Leu/-Trp/-His/-Ade/X-α-Gal/AbA (QDO/X/A) plates. Plasmids from the final positive clones were isolated and amplified by transformation into *Escherichia coli* DH5α competent cells. To confirm the interaction, candidate clones were cotransformed with the GRHL2 bait plasmid into Y2H Gold yeast strain and grown on QDO/X/A selective media. Finally, the identity of prey constructs was determined by DNA sequencing and NCBI BLAST search.

### Cell culture, transfections, and retroviral infections

MDA-MB-231, T47-D, and ZR75-1 breast cancer cells and COS-7 cells were cultured as described elsewhere (6). All cell lines were authenticated by short tandem repeat profiling. Transient transfection of COS-7 cells with plasmid DNA was performed using Lipofectamine 3000 reagent (Invitrogen) according to the manufacturer’s instructions. Retroviral infections of MDA-MB-231 and COS-7 cells using the pMXs-IP retroviral expression vector encoding WT or mutant GRHL2 proteins was conducted as described previously (6). Infected cells were selected with 0.5 μg/ml (MDA-MB-231) or 2 μg/ml (COS-7) puromycin. Expression of GRHL2 mRNA and proteins was verified confirmed by qRT-PCR and Western blot analysis.

### Plasmid constructions

Expression plasmids were generated by RT-PCR amplification using Q5 High-Fidelity DNA Polymerase (New England BioLabs) and insertion of PCR products into corresponding expression vectors following standard procedures (41). All mutants were generated using a modified quick-change site-directed mutagenesis protocol (Stratagene). Identities and functionality of all expression plasmids were verified by DNA sequencing and Western blot analysis. For transient transfections of COS-7 cells, the GRHL2 cDNA was RT-PCR amplified from T47-D cells with oligonucleotides 5′-ATTGGATCAAACATGTCA-CAAGAGTCGG-3′ and 5′-CTAGATTTCCATGAGCGTGACCTTGAAGC-3′ was inserted into the mammalian expression vector pCMX3b-FLAG in-frame with the coding sequence of the FLAG epitope yielding pCMX3b-GRHL2 (WT). A number of GRHL2 cDNAs coding mutant GRHL2 proteins were generated and yielded plasmids pCMX3b-FLAG-GRHL2 (K159R), pCMX3b-FLAG-GRHL2 (K205R), pCMX3b-FLAG-GRHL2 (K366R), pCMX3b-FLAG-GRHL2 (K424R), pCMX3b-FLAG-GRHL2 (K453R), pCMX3b-FLAG-GRHL2 (K531R), pCMX3b-FLAG-GRHL2 (K556R), pCMX3b-FLAG-GRHL2 (E161A), pCMX3b-FLAG-GRHL2 (T164A), and pCMX3b-FLAG-GRHL2 (T164D). In some immunofluorescence stainings, the pCMX3b-GRHL2 (WT) expression construct was replaced by an expression plasmid pSF-1-GRHL2 (WT) encoding influenza hemagglutinin epitope (HA)-tagged GRHL2. For coimmunoprecipitation experiments, the GRHL2 cDNA was inserted into the pEF-IRES-P-Strep-Tag vector downstream of the Strep-Tag coding sequence to generate the pEF-IRES-P-Strep-Tag-GRHL2 plasmid. cDNA encoding activated, HA-tagged SUMO-1 or SUMO-2 were generated by RT-PCR amplification and were inserted into the phCMV2 vector yielding phCMV2-SUMO-1 (GG) and phCMV2-SUMO-2 (GG). Plasmids encoding activated, His_6_-tagged SUMO-1 or SUMO-2 were generated by RT-PCR amplification and were inserted into the phCMV4 vector to create phCMV4-SUMO-1 (GG) and phCMV4-SUMO-2 (GG). Expression constructs encoding WT, full-length SENP-1, SENP-2, SENP-3, SENP-5, SENP-6, and SENP-7 were generated by RT-PCR amplification from T47-D cells and were introduced into pCMX3b-FLAG vector in-frame with the coding sequence of the FLAG epitope. Plasmids coding for mutant SENP-1 and SENP-2 proteins were generated by site-directed mutagenesis and yielded pCMX3b-FLAG-SENP1 (C630S) and pCMX3b-FLAG-SENP2 (C548S). The cDNAs encoding full-length, HA-tagged PIAS1-4 proteins were created by RT-PCR amplification from T47-D cells and were ligated into the phCMV3 vector to generate phCMV3-PIAS1, phCMV3-PIAS2, phCMV3-PIAS3, and phCMV3-PIAS4. To obtain the GST-GRHL2 (T118-R209) and GST-ATF2 (M19-L114) fusion proteins, the corresponding regions were PCR-amplified and inserted into the pGEX-3X vector. cDNA fragments encoding the corresponding mutant forms of GRHL2 (GST-GRHL2 (T164A)) or ATF2 (GST-ATF2 (T69A, T71A)) were created by site-directed mutagenesis. For retroviral gene transfer, the expression plasmids pMXs-IP-GRHL2 (WT), pMXs-IP (K159R), pMXs-IP (E161A), and pMXs-IP-GRHL2 (T164A) were generated by subcloning the corresponding cDNAs into pMXs-IP retroviral vector. The bait plasmid for the yeast two-hybrid screen was created by inserting a PCR-amplified fragment encoding full-length human GRHL2 into pGBKT7 plasmid in-frame with the GAL4 DNA binding domain. cDNAs encoding PIAS1-4 proteins were ligated into pGADT7 vector in-frame with the GAL4 activation domain. Plasmids pGBKT7-53, pGBKT7-Lam, and pGADT7-T were purchased from Clontech. Expression plasmids encoding WT, CA, or kinase-dead MKK6 or MKK7 proteins were kindly provided by Roger Davies through Addgene (42, 43).

### Antibodies and reagents

The generation and specificity of the polyclonal anti-GRHL2 antibody was comprehensively described previously (2, 6). Antibodies specific for HA- and FLAG-tags, PSPC1/PSP-1 (N-terminal) (all from Sigma), tubulin (11H10), SUMO-1, SUMO2/3 (18H8), p38 MAPK, phospho-p38 MAPK (Thr180/Tyr182) (28B10), phospho-Thr-Pro (P-Thr-Pro-101), HSP70 (6B3), ubiquitin (P4D1), fibrillarin (C13C3), His-tag (all from Cell Signaling Technology), E-cadherin (clone 36), SC-35 (both from BD Biosciences), PSMB5 (Thermo Fisher Scientific), CD-24 (EPR3006(N)) (Abcam), EZH2 (Diagenode), fibrillarin (38F3) (antibodies-online.com), promyelocytic leukemia (PG-M3) (Santa Cruz), and GST-Tag (Proteintech). Secondary antibodies used for Western blot analysis include anti-rabbit IgG, HRP-linked, and anti-mouse IgG, HRP-linked antibodies (both from Cell Signaling). For indirect immunofluorescence analyses, a range of distinct fluorescent dye (Alexa Fluor 488 or 555)–conjugated secondary antibodies against rabbit, mouse or rat IgG (H + L) were used (all from Thermo Fisher Scientific). NEM, MG-132, and CHX were purchased from Sigma. Inhibitors SB203580 and SP600125 were obtained from InvivoGen and Santa Cruz, respectively.

### Western blot analysis

Whole-cell extracts from cultured cells were prepared using lysis buffer containing 1% NP-40, 0.5% Na-DOC, 0.1% SDS, 150 mM NaCl, 20 mM sodium phosphate (pH 7.4), 25 mM NEM, and protease/phosphatase inhibitor cocktails (Sigma). Following incubation on ice for 30 min, supernatants were collected by centrifugation, mixed with 2X SDS sample buffer, and denatured at 95 to 100 °C for 5 to 10 min. Samples were separated on denaturing polyacrylamide gels (44) and electroblotted onto polyvinylidene fluoride membranes (Sigma) by semidry transfer. Membranes were blocked in 5% milk powder in Tris buffered saline with Tween 20 (TBS-T) (137 mM NaCl, 0.05% Tween-20, 20 mM Tris/HCl, pH 7.6) for 1 h at room temperature (RT) and incubated with primary antibodies diluted in 5% bovine serum albumin (BSA) in TBS-T overnight at 4 °C. Membranes were then washed 3 × 5 min in TBS-T and incubated with secondary antibody diluted in 5% skim milk powder in TBS-T for 1 h at RT. Following three washes for 5 min in TBS-T, signals were detected using the enhanced chemiluminescence.

### Immunofluorescence staining

Cells grown in 4-well chamber slides (Corning) were washed twice with PBS and were then fixed for 15 min in 4% paraformaldehyde at RT. Cells were washed three times with PBS and were permeabilized using 0.2% Triton X-100 (Sigma) for 5 min at RT. Following three washes with PBS, nonspecific binding sites were blocked by incubation for 30 min in blocking buffer (1% BSA in PBS). Subsequently, cells were incubated with the primary antibody followed by the corresponding fluorescent dye–conjugated secondary antibody (dilution 1:200) (Thermo Fisher Scientific) in blocking solution, for 90 min at RT each. After each individual antibody incubation, cells were washed four times with PBS, counterstained with 4′,6-diamidino-2-phenylindole (Sigma), and were finally mounted in Mowiol (Sigma). Microscopic analysis was performed using an Axioplan2 microscope equipped with AxioVision SE64 (Re. 4.9.1) software (Zeiss). In some cases, cells were fixed with −20 °C methanol for 15 min on ice, rehydrated with PBS for 5 to 10 min at RT, and were then processed as described above. The pre-extraction protocol involved treatment of cells with 0.5% (w/v) Triton X-100 in PBS for 2 min at RT. Following one wash with PBS, cells were fixed with 4% paraformaldehyde in PBS for 15 min at RT and processed as described above.

### Luciferase reporter assays

COS-7 cells cells were transiently cotransfected with 0.25 μg of GRHL2 expression plasmids, 0.25 μg of a reporter plasmid containing five copies of the GRHL2 consensus binding site (AACCGGTT) upstream of a minimal promoter and the Firefly luciferase (*luc2*) gene, and 5 ng of the pGL4.74 normalization plasmid encoding the Renilla luciferase (*hRluc*) (Promega) using Lipofectamine 3000. For Rab25 promoter assay, the human Rab25 promoter (≈1600 bp) amplified from genomic DNA using primers Rab25-F1 (5′-GCGCATCCTCGAGTGTCAAGGAAGGGCAGAAGT-3′) and Rab25-R1 (5′-GCGCATCAAGCTTAGAGGAC-GGAAGCTGAGAAC-3′) and inserted into the XhoI/HindIII-sites of the pGL4.10 vector (Promega) was used. In some cotransfection experiments, equal amounts (0.17 μg) of GRHL2 expression plasmids, reporter plasmid, and an expression plasmid encoding HA-tagged SUMO-1 and the normalization plasmid (5 ng) was used. Forty-eight hours posttransfection, cell extracts were prepared using passive lysis buffer (Promega). Samples were assayed in triplicate for luciferase activities using a Dual luciferase assay kit (Promega) with the GloMax Discover Multimode plate reader (Promega). Before calculating the fold activation value, luciferase activity of each sample was normalized with respect to the activity of Renilla luciferase. Unless stated otherwise, luciferase activity from COS-7 cells transfected with GRHL2 (WT) was set arbitrarily at 1 for calculation of fold activation. In some experiments, COS-7 cells were transiently cotransfected with expression plasmids encoding GRHL2 (WT) proteins (100 ng), increasing amounts of PIAS1-4 proteins (0, 50, 100, and 150 ng), the pGL4.23-5xCS reporter plasmid (250 ng), and the pGL4.74 normalization plasmid (5 ng). The total amount of expression plasmid was kept constant by addition of the corresponding vector without insert.

### Pull-down assay

COS-7 cells cotransfected with GRHL2 and His_6_-tagged SUMO-1 or SUMO-2 expression plasmids were harvested and resuspended in buffer A (6 M guanidine/HCl, 10 mM imidazole, 0.1 M Na_2_HPO_4_/NaH_2_PO_4_, pH 8.0). Lysates were sonicated, precleared by centrifugation, and added to 50 μl of Ni-NTA agarose (QIAGEN) pre-equilibrated in buffer A. Following incubation at RT for 4 to 6 h, beads were successively washed with buffer A, buffer A/TI (1 volume of buffer A, 3 volumes. of buffer TI [20 mM imidazole, 25 mM Tris/HCl, pH 6.8]), and buffer TI. After centrifugation for 10 s at top speed at RT, the supernatant was discarded. Any residual buffer trapped in agarose beads was removed using a 26 G needle. Bound proteins were released from the matrix by adding 50 μl of 2× SDS sample buffer and boiling at 95 to 100 °C for 10 min. The supernatant was harvested after centrifugation at top speed for 10 min at RT and was then subjected to Western blot analysis.

### Coimmunoprecipitation assays

COS-7 cells were transiently cotransfected with expression plasmids encoding GRHL2 proteins with an N-terminal Twin-Strep-Tag and HA-tagged PIAS or FLAG-tagged SENP proteins. Forty-eight hours posttransfection, cells were lysed in co-immunoprecipitation buffer containing 50 mM Tris/HCl (pH 8.0), 300 mM NaCl, 0.4% NP-40, 10% glycerol, and protease inhibitors for 30 min at 4 °C. Samples were cleared by centrifugation, and supernatants were collected and diluted with an equal volume of dilution buffer (0.4% NP-40, 50 mM Tris/HCl, pH 8.0, supplemented with protease inhibitors). Immunoprecipitation was then performed by incubating the lysate (1 mg) with 1 μl pre-equilibrated MagStrep “type 3” XT (IBA Lifesciences) or 10 μl anti-Flag magnetic beads (Selleckchem) for 4 h at 4 °C under gentle rotation. Beads were then washed five times with washing buffer (150 mM NaCl, 0.4% NP-40, 5% glycerol, 50 mM Tris/HCl, pH 8.0). Finally, protein complexes were eluted from beads with Laemmli sample buffer and then were subjected to Western blot analysis.

### qRT-PCR analysis

Differential mRNA expression was analyzed following extraction of total RNA from cells and reverse transcription using First-Strand cDNA Synthesis kit (Thermo Fisher Scientific) and random hexamers. First-strand reverse transcribed cDNA was then diluted 1:20 in water before use in real-time PCR. PCRs were performed using the Thermo Scientific Maxima SYBR Green/Rox qPCR Master Mix (2x) (Thermo Fisher Scientific) in a CFX96 Real-Time System equipped with C1000 Touch Thermal Cycler and CFX3.1 software (Bio-Rad, https://www.bio-rad.com/de) according to the manufacturer’s instructions. Real-time PCR data analysis was performed using the ΔΔCT method with RPLP0 as endogenous reference. The following primers were used: GRHL2 (5′-CATGCCTGATCTCCACTCACAG-3′ and 5′-CTGCCACCTTCTCGTTCATCA-3′), CD24 (5′-CACTGCTCCTACCCACGCAGAT-3′ and 5′-CTTGGTGGTGGCATTAGTTGG-3′), CDH1 (5′-CAGGAACCTCTGTGATGGAG-3′ and 5′-CACTGATGACTCCTGTGTTCCTG-3′), and RPLP0 (5′-ACCCAGCTCTGGAGAAACTGC-3′ and 5′-TGAGGTCCTCCTTGGTGAACA-3′).

### Generation of polyclonal phospho-GRHL2 (T164) antibody

Polyclonal phospho-GRHL2 (T164) antibodies were raised by immunizing rabbits with the phosphorylated peptide VKAEDF(pT)PVFMAPP of human GRHL2 conjugated with KLH. Antibodies were affinity-purified from antisera using immobilized phosphopeptide (positive selection), followed by depletion of the nonphosphospecific fraction against the unmodified peptide (negative selection). The eluate was collected and concentrated. The initial validation for phospho-specificity of cross-affinity purified antibodies was performed by indirect ELISA.

### Protein half-life measurements

COS-7 cells were transiently transfected with GRHL2 expression plasmids and HA-tagged SUMO-1 and cultured for 48 h. After treatment with CHX for the indicated times at a final concentration of 50 μg/ml, cells were harvested and cleared lysates were subjected to Western blot analysis. Quantification of Western blot signals at nonsaturating exposures was performed using ImageJ software (45).

### *In vitro* protein kinase assay

GST fusion proteins including GST alone, GST-GRHL2 (T118-R209) (WT and T164A mutant), and GST-ATF2 (M19-L114) (WT and T69A, T71A double-mutant) were expressed in *E. coli* and purified with GSH-agarose beads using standard protocols (41). *In vitro* kinase assays were performed in 25 μl of kinase buffer (5 mM β-glycerophosphate, 2 mM DTT, 0.1 mM Na_3_VO_4_, 10 mM MgCl_2_, 25 mM Tris/HCl, pH 7.5) supplemented with 200 μM ATP, 40 ng/ml of purified, recombinant protein, and 12.5 ng/μl activated p38α MAPK (ProQinase) for 30 min at 30 °C. Reactions were terminated by the addition of 2× SDS sample buffer and subsequently analyzed by SDS-PAGE and immunoblotting.

### Protein structure and SUMOylation site predictions

AlphaFold2 (https://alphafold.ebi.ac.uk/) was used for predicting the overall structure of GRHL2 (46). The presence of intrinsically disordered regions and disorder content in GRHL2 was predicted using the following servers: DISOclust (http://www.reading.ac.uk/bioinf/IntFOLD/) analyzes the per residue structural variation across the 3D models generated by the IntFOLD server (47). PrDOS (https://prdos.hgc.jp/cgi-bin/top.cgi) predicts natively disordered regions from the amino acid sequence (48). PONDR-Fit (http://original.disprot.org/pondr-fit.php) combines the outputs of several individual disorder predictors (49). DisoPred 3.1 (http://bioinf.cs.ucl.ac.uk/psipred/) represents a machine learning–based approach for the detection of intrinsically disordered regions (50). SUMOylation prediction tools include JASSA (51), GPS-SUMO 2.0 (52), and SUMO Plot (http://www.abgent.com/sumoplot).

### Immunohistochemistry

Immunohistochemical analysis of GRHL2 expression on a high-density breast cancer prognosis tissue microarray containing formalin-fixed, paraffin-embedded primary breast tumor specimens with available clinical follow-up and histopathological data was described previously (6). Briefly, tissue sections were deparaffinized and then were subjected to pressure cooker pretreatment in citrate buffer (BioGenex) for 5 min at 125 °C. After blocking of endogenous peroxidase activity using DAKO REAL peroxidase blocking solution (Dako), the polyclonal anti-GRHL2 antibody (1:500 dilution) was applied overnight at 4 °C. Subsequently, slides were incubated with peroxidase-labeled EnVision polymer coupled with goat anti-rabbit/mouse immunoglobulins (Dako) for 15 min at RT. GRHL2 expression was visualized using 3,3′-diaminobenzidine as chromogen in substrate buffer containing hydrogen peroxide. Finally, sections were counterstained with Mayer’s hemalum solution (Merck) and permanently mounted. Irrespective of staining intensity, nuclear GRHL2-specific immunoreaction was classified as negative (0), diffuse (D), granular (G), or mixed (M). Statistical analyses were performed with the program R version 4.4.1 and with the R-packages effects version 4.6.0 and survival version 3.8.3. Associations of nuclear distribution patterns with histopathological variables were examined using logistic regression models. For estimation of disease-specific and overall survival, Kaplan–Meier plots were used, and statistical significance was analyzed by univariate log-rank tests. Multivariate cox regression models with stepwise backward selection of the covariates were used to determine the parameters with greatest influence on survival. *P* values of <0.05 were considered statistically significant.

### Statistical analysis

Experiments were repeated at least three times in triplicate unless stated otherwise. All quantitative data are presented as mean values (±SD). Unpaired two-tailed Student’s *t* test was used for performing analysis of variance in EXCEL software (https://microsoft.com). A *P* value of 0.05 or less was considered statistically significant.

## Data availability

Other data supporting the study findings or any additional information required are available from the corresponding author upon reasonable request.

## Supporting information

This article contains supporting information.

## Conflict of interest

The authors declare that they have no conflicts of interest with the contents of this article.
