# Supplementary material for: The activity of the EMT suppressor GRHL2 is regulated by SUMOylation
Source: J Biol Chem. 2025 Aug 30;301(10):110662. doi: 10.1016/j.jbc.2025.110662 (PMC12495329; doi:10.1016/j.jbc.2025.110662)
Supplement: Supporting Figures and Table [file mmc1.pdf]

SUPPORTING INFORMATION

The activity of the EMT suppressor GRHL2 is regulated by SUMOylation

Sonja Santjer[1], Yang Xu[1], Sabine Riethdorf[1], Gerhard Schön[2], Johanna Neu[1], Klaus Pantel[1], and Volker Assmann[1#]

From the [1]Institute of Tumor Biology, University Medical Center Hamburg-Eppendorf, Hamburg, Germany; [2]Institute for Medical Biometry and Epidemiology, University Medical Center Hamburg-Eppendorf, Hamburg, Germany.

Supporting Table S1

Supporting Figure S1

Supporting Table S1

Subnuclear distribution of GRHL2 immunostaining in correlation to histopathological characteristics of breast cancer tissue samples

	Subnuclear distribution of GRHL2 immunostaining				
	**All cancers (N)**	**Negative (N)**	**Diffuse (D)**	**Granular (G)**	**Mixed (M)**
**Total number**	***1075***	***29 (2.7%)***	***431 (40.09%)***	***70 (6.51%)***	***545 (50.7%)***
**Histological type**					
Ductal carcinoma*	**780**	20	295	56	409
Lobular carcinoma	**131**	1	84	2	44
Other types	**164**	8	52	12	92
**Tumor size**					
pT1	**363**	5	164	18	176
pT2	**526**	15	198	37	276
pT3	**64**	4	27	6	27
pT4	**122**	5	42	9	66
**Lymph node status**					
pN0	**532**	11	226	30	265
pN1/2	**543**	18	205	40	280
**Histological grade**					
G1	**272**	8	131	11	122
G2	**414**	9	194	21	190
G3	**389**	12	106	38	233
**Mitotic index**					
M1	**537**	13	268	21	235
M2	**197**	7	75	13	102
M3	**341**	9	88	36	208
**ER status**					
Positive	**821**	13	365	33	410
Negative	**254**	16	66	37	135
**PR status**					
Positive	**372**	3	174	12	183
Negative	**703**	26	257	58	362

*Invasive carcinoma of no special type (NST; WHO 2012); ER: estrogen receptor; PR: progesterone receptor

Supporting Figure S1

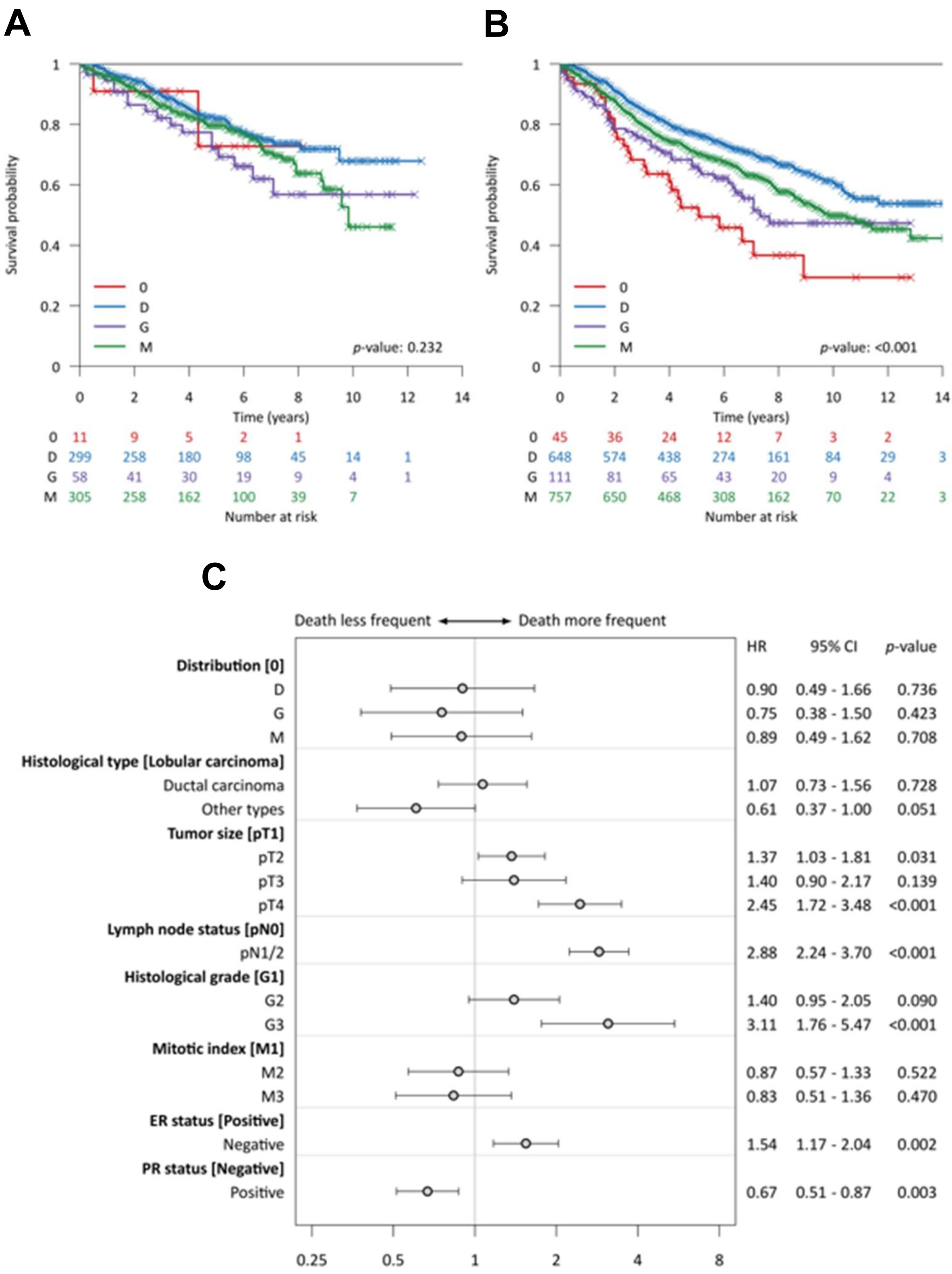

Supporting Figure S1. Prognostic relevance of GRHL2 in breast cancer. *A* and *B*, Kaplan-Meier survival curves for prognostic relevance of subnuclear GRHL2 distribution patterns (*A*, disease-free survival; *B*, overall survival). *C*, Multivariate cox regression analysis of survival visualized as forest plot. Subnuclear distribution of GRHL2 immunostaining was classified as negative (0), diffuse (D), granular (G), and mixed (diffuse and granular) (M). OR, odds ratio; CI, confidence interval; ER, estrogen receptor; PR, progesterone receptor.
